# Exploring Spiders Without Venom as New Sources of Peptidase Inhibitors

**DOI:** 10.3390/ijms27010186

**Published:** 2025-12-24

**Authors:** Jefferson O. Silva, Ana Carolina O. Silva, Rodrigo Valladão, Oscar Bento Neto, Vinicius Carius de Souza, Clelia Ferreira, Walter Ribeiro Terra, Adriana Rios Lopes

**Affiliations:** 1 Biotechnology Postgraduate Program, Institute of Biomedical Sciences, University of São Paulo, 2415 Professor Lineu Prestes Avenue, São Paulo 05508-000, Brazil; jefferson.o.s@alumni.usp.br (J.O.S.); ana.carolina@icb.usp.br (A.C.O.S.); 2 Biochemistry Laboratory, Butantan Institute, 1500 Vital Brazil Avenue, São Paulo 05503-900, Brazil; rvalladao@usp.br (R.V.); oscar.bentoneto@gmail.com (O.B.N.); 3 Institute of Chemistry, Department of Biochemistry, University of São Paulo, 748 Professor Lineu Prestes Avenue, São Paulo 05508-000, Brazil; clfterra@iq.usp.br (C.F.); warterra@iq.usp.br (W.R.T.); 4 Special Laboratory of Applied Toxinology, Butantan Institute, 1500 Vital Brazil Avenue, São Paulo 05503-900, Brazil

**Keywords:** peptidase, inhibitors, spiders, structure, digestion, Uloboridae, transcriptome

## Abstract

Peptidases constitute at least 2% of genes in living organisms and participate in nearly all physiological processes across life forms. Conversely, peptidase inhibitors are essential for regulating proteolytic activity and have been widely applied. Combining high-throughput sequencing of novel peptidase inhibitor sources with molecular modeling and drug design currently represents an efficient strategy for developing new molecules. Venomous spiders harbor a wide array of peptidase inhibitors in both their venom and digestive system. However, biochemical and transcriptomic investigations of non-venomous spiders (Uloboridae) remain recent and scarce. Here, transcriptomic and biochemical analyses of the Uloboridae spider *Zosis geniculata*’s digestive midgut diverticula (MD) revealed that this species exhibited a digestive enzyme profile similar to that of other spiders. Furthermore, the MD transcriptome identified 19 peptidase inhibitors belonging to six inhibitor families. Serine peptidase inhibitors were the most abundant and diverse, while metallopeptidases represented the main proteolytic enzymes, suggesting that these inhibitors may have evolved to counteract prey-derived peptidases. Inhibitory assays using trypsin from potential insect prey confirmed this activity. The diversity and abundance of these molecules highlight Uloboridae spiders as promising novel sources of proteolytic inhibitors.

## 1. Introduction

In the co-evolutionary process between peptidases and inhibitors, a myriad peptidase inhibitors have been selected and are effective in regulating the most diverse physiological processes in which proteolytic enzymes are involved. The peptidases are among the most varied and expressed genes in all living beings [1]. The search for new peptidase inhibitors is ongoing due to the deleterious processes they mediate: from human diseases to pest control in plants and animals. Thus, the search for new inhibitory molecules is mandatory. 

The development of new peptidase inhibitors requires characterizing enzyme active sites through kinetic and structure-activity relationship analyses to design effective and selective molecules that minimize off-target effects [2]. Nature has spent billions of years selecting the interaction of enzyme active sites and their respective inhibitors. The literature shows that natural inhibitors are usually safer, with a specific mechanism of action that leads to fewer off-target effects. This is a desirable trait for developing novel molecules. Given this, a continuous search for bioactive inhibitors has been exploring the enormous potential of underexplored natural sources, helped by the technical improvements of the last decade in protein and nucleic acid sequencing.

Spiders are, after insects, the largest taxonomic group of terrestrial arthropods and occupy most ecological niches of our planet [3]. This involves exposure to and acquisition of different diets within each spider group, and distinct selective pressures acting on the evolution of peptidases/inhibitors [4]. Spider venoms have been explored as a source of peptides with pharmacological effects [5]. In the last decade, biochemical data, combined with transcriptomic and proteomic data on Arachnida and, particularly, on spider digestive systems, have shown that, besides the venom gland, the digestive system is also a rich source of peptidase inhibitors [6,7,8]. The main inhibitor families in spiders, according to the MEROPS classification, are Kunitz, TIL, Kazal, serpins, and cystatins. Uniprot has deposited 600 spider sequences of spider Kunitz inhibitors; 829 TIL spider inhibitors; 545 Kazal spider inhibitors; 451 spider serpins and 94 spider cystatins. The Kunitz domain is typically composed of a single peptide chain of approximately 60 amino acid residues, stabilized by three disulfide bridges, and is present in both I02 and I03 Merops inhibitor families [9,10]. The Kunitz domain is typically composed of a single peptide chain of approximately 60 amino acid residues, stabilized by three disulfide bridges, and is present in both I02 and I03 Merops inhibitor families [9,10]. Trypsin Inhibitors-Like (TIL), described initially from *Ascaris suum*, are clustered at Merops family I8 and are typical small proteins stabilized by disulfide bonds. Usually, these molecules are inhibitors of serine S1 peptidases and some metallopeptidases. Kazal (KPIs) belongs to the I1 MEROPS family [11,12] characterized by a general amino acid sequence: CXₐ-CX_b_-PVCG-X_c_-YX_d_-CX_e_-CX_f_-C, where the subscripts a, b, c, d, e, and f represent numbers of residues. Although some amino acids within this motif are relatively conserved, most exhibit high variability both within and between different invertebrate species.

Uloboridae spiders belong to the Araneomorphae infraorder and the Deinopoidea superfamily. Deinopoidea and Aranoidea together form the large clade of Orbicularie [13]. Uloboridae have lost their venom glands throughout evolution. Their preying behavior depends exclusively on prey wrapping [14] and the use of digestive fluid. However, Valladao and co-authors, as well as Peng and co-authors, have shown that even Uloboridae spiders did not lose their toxicity, as many typical spider venom components were identified in their midgut diverticula (MD), such as phospholipase D [14,15]. 

**Figure 1:**
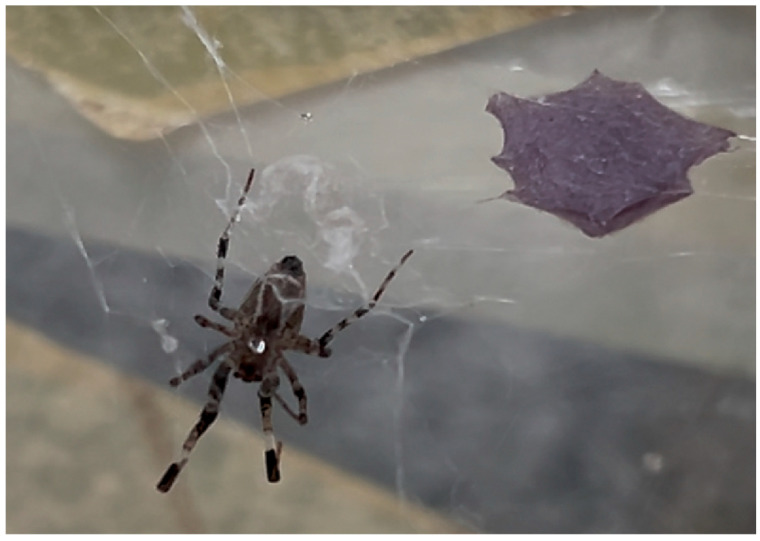
Adult *Z. geniculata* and ootheca. The image shows an adult female of *Z. geniculata* suspended on its web near the ootheca. The ootheca is covered by silk and attached to the substrate, suggesting active reproductive behavior. The morphological features of the female, including long striped legs and a flattened, elongated opisthosoma, are characteristic of this species. Photo by Adriana Rios Lopes.

*Zosis geniculata* (Uloboridae) (Figure 1) has a pantropical distribution and is frequently found in buildings near human dwellings [16]. Considering the scarcity of studies involving non-venomous spiders and the unexplored potential of their digestive system as a source of active biomolecules, this work aimed to: (a) biochemically characterize the digestive enzymes present in the MD of *Z. geniculata* in comparison to other spiders, and (b) assemble and identify, through transcriptomic analysis, the peptidase inhibitors expressed in this tissue, (c) use the most expressed inhibitory sequences in molecular modeling analysis to evaluate molecular composition and biotechnological potential of this species’ digestive system.

## 2. Results

### 2.1. Determination of Specific Activities in the Digestive System of Z. geniculata

**Figure 2:**
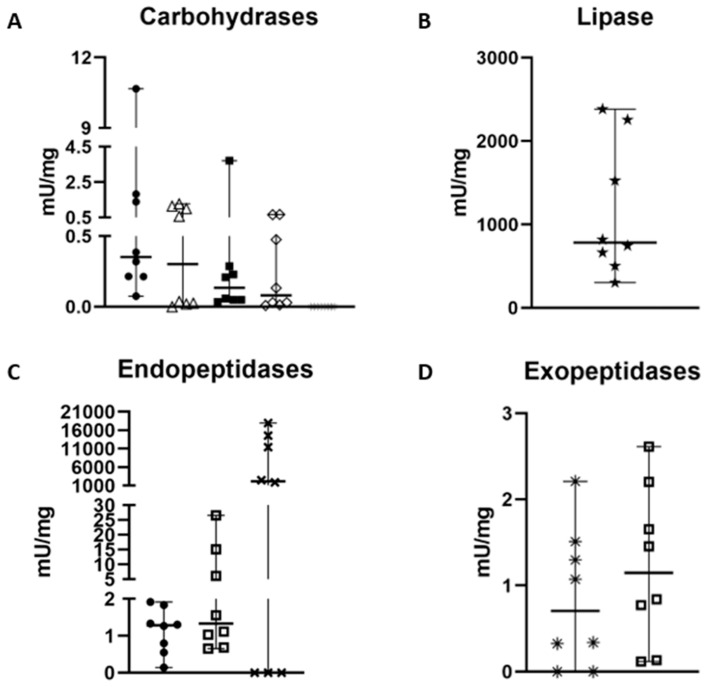
Specific activity measurement of the 11 main enzymes (mU/mg except astacin fluorescent unit.min^−1^. mL^−1^) involved in the digestion process of *Zosis geniculate*. (**A**) chitinase is represented by •, fucosidase by △, hexosaminidase by ◼, mannosidase by ♢ and amylase by *. (**B**) Lipase is represented by ★. (**C**) Cathepsin L is represented by •, while trypsin is represented by ☐ and astacin by _✖_. (**D**) Aminopeptidase is represented by *, and carboxypeptidase is represented by ☐. Graphs were generated in GraphPad.

The samples showed an average protein concentration of 2.33 ± 0.25 µg/µL, corresponding to 0.58 mg of protein per MD. Among the measured carbohydrases, specific activities were detected for chitinase (1.88 ± 0.77 mU·mg^−1^), followed by hexosaminidase (0.69 ± 0.27 mU·mg^−1^), α-L-fucosidase (0.50 ± 0.17 mU·mg^−1^), and α-mannosidase (0.25 ± 0.09 mU·mg^−1^) (Table 1, Figure 2). α-Amylase showed no activity with either glycogen or starch as substrate. The class of exopeptidases, represented by carboxypeptidase and aminopeptidase, exhibited activities of 1.22 ± 0.26 mU·mg^−1^ and 1.12 ± 0.19 mU·mg^−1^, respectively. Among the endopeptidases, astacin showed 96.15 ± 39.33 fluorescent units min^−1^·mg^−1^, trypsin 6.61 ± 2.51 mU·mg^−1^, and cathepsin-L 1.14 ± 0.17 mU·mg^−1^. Lipase activity was 1150.3 ± 239.9 mU·mg^−1^ (Figure 2). 

### 2.2. Inhibition Assays of Different Trypsins by Preparations from Z. geniculata

**Figure 3:**
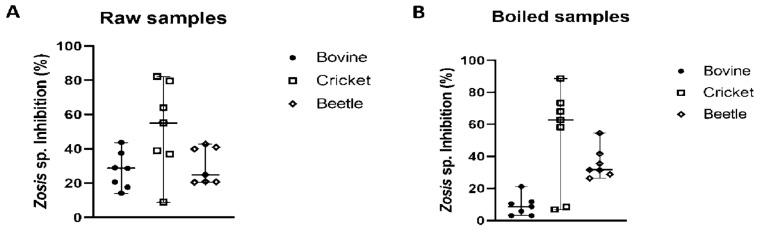
Inhibitory activity of *Z. geniculata* samples against bovine trypsin, *Gryllus sp*. trypsin and *Z. morio* trypsin. In graphs (**A**,**B**), the inhibition percentages using *Z. geniculata* samples as an inhibitor against bovine, cricket and beetle trypsin activity are presented. The median is represented by the line in the middle. The poinsts referring to bovine trypsin are represented by, while cricket trypsin is represented by and beetle trypsin by. Graphs were generated in GraphPad.

The median percentage of inhibition of *Gryllus* sp. trypsin activity by non-heated *Z. geniculata* MD homogenate was 55%, followed by the median percentage of inhibition of bovine trypsin activity at 29%, and inhibition of *Z. morio* trypsin activity at 25%. Statistically, the median values of the three groups (cricket, beetle, and bovine trypsin) analyzed did not vary significantly (*p* < 0.05; *p*-value = 0.0705). However, there was a significant difference between the two groups: cricket and beetle, according to Tukey’s multiple comparison test. For heated *Z. geniculata* samples, the median percentage of inhibition of cricket trypsin activity was 63%, followed by the median inhibition of bovine trypsin at 9%, and the median inhibition of *Z. morio* trypsin at 32%. In the boiled *Z. geniculata* samples, the median values of the three groups (bovine, cricket, and beetle trypsin) differed significantly among them (*p* < 0.05; *p*-value < 0.0001) (Figure 3). In this case, the difference between the bovine and cricket groups was significant.

### 2.3. RNA Extraction, Transcriptomic Analysis, and Identification of Inhibitors from Z. geniculata

RNA extraction was performed on three different MD samples from *Z. geniculata*, yielding concentrations of 2.6 μg/μL, 45 ng/μL, and 20 ng/μL, generating 13,622,353, 16,095,243, and 17,554,506 PE reads, respectively. The transcriptome was assembled using trimmed paired-end reads totaling 33,412,524. De novo assembly produced 59,143 contigs. After translating the transcripts, considering only sequences with a predicted domain, and removing duplicate isoforms, the final transcript set comprised 13,948 unigenes, achieving BUSCO completeness scores of 79.3% for the Arachnida dataset and 91% for the Eukaryota dataset. 

Among these contigs, digestive enzymes were identified, and their expression was analyzed using TPM values (Table 2). Amidst the carbohydrases, chitinase showed the highest TPM value, while exopeptidases are mainly represented by carboxypeptidase. Astacin exhibited the highest TPM values for endopeptidases. Notably, among the most expressed sequences, 47 toxin-related sequences with distinct domains were identified by homology analysis using Pfam databases (Table 3).

### 2.4. Peptidase Inhibitor’s Structure 

**Figure 4:**
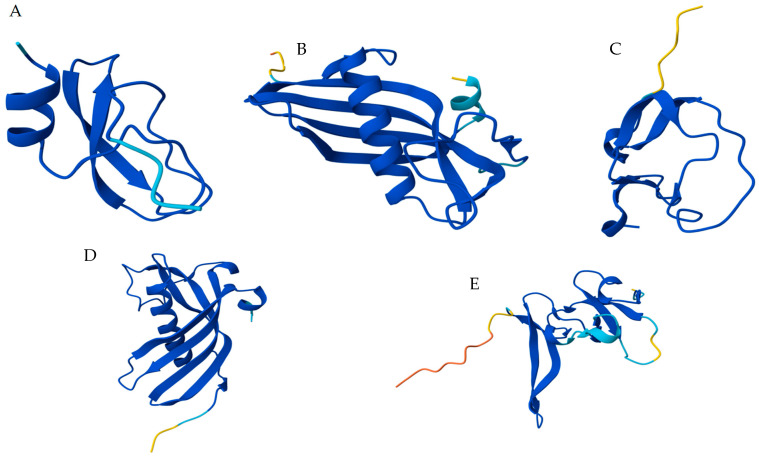
Molecular modeling of the main peptidase inhibitors identified at MD transcriptome from *Z. geniculata*. (**A**) Kunitz domain present at papilin (contig DN808)—serine peptidase inhibitor with positive amino acid residue at the reactive center; (**B**) Cystatin (contig DN485); cysteine peptidase inhibitors which commonly present glycine residues at P1 position; (**C**) TIL (contig DN474); (**D**) Serpin (Contig DN211); (**E**) Atracotoxin (Contig DN746). TIL, Serpin, and Atracotoxin are serine peptidase inhibitors and usually contain positively charged residues at P1 in their loop-located reactive sites. Proteins were modeled using AlphaFold3 and are colored according to pIDDT/pLDDT scores, where dark blue represents the higher-confidence regions and yellow the lower ones. Model Ramachandran plots are in the Supplementary Data.

Nineteen distinct sequences of peptidase inhibitors were identified at the MD from *Z. geniculata*, representing Kunitz > Cystatin > TIL> Serpin > FXa_inhibition > Kazal (Table 4). Besides that, seven sequences structurally related to the atracotoxin family were identified in the *Z. geniculata* transcriptome (DN746, DN1389, DN2267, DN3333, DN872, DN650, and DN10402), with varying expression levels and reported as toxins (Table 3). Modeling of the most expressed sequences revealed distinct structural profiles of serine peptidase inhibitors in spiders (Figure 4).

## 3. Discussion

In this work, the advancement of the molecular study of Uloboridae through the characterization of *Z. geniculata* MD through biochemical activities, enzymatic inhibition, and transcriptome analysis has evidenced similarities and differences between Uloboridae spiders and other spider families. *Uloborus diversus* is the top hit species in sequence identities in blastp analysis comparisons, followed by *Stegodyphus dumicola*, *Argiope bruennichi*, *Parasteatoda tepidariorum*, *Centruroides sculpturatus*, and *Centruroides vittatus*. This data shows that *Z. geniculata* shares a large portion of its MD contigs with species from the same family, but also shares many proteins with spiders that possess venom glands and with other arachnids such as the scorpion *Centruroides* sp., suggesting that the production of digestive enzymes, toxin-like proteins and peptidase inhibitors by the MD is rather an older process along Arachnida evolution than an adaptation selected in Uloboridae.

### 3.1. The Evolution of Digestion in Spiders

The transcriptome data obtained from *Z. geniculata*, as well as the first MD transcriptome and the digestive fluid proteomic analysis of *Nephilingis cruentata* (GEWZ00000000.1), the opisthosoma transcriptome of *Acanthoscurria geniculata* and the proteomic analysis of the digestive fluid of *Acanthoscurria geniculata* and *Stegodyphus dumicola* offer valuable insights into the evolution of the digestive process in spiders [7]. It is important to note that additional efforts in sequencing specific tissues are necessary to fully understand the various physiological systems in spiders, since the most recent genome and transcriptome data have focused primarily on genes involved in silk and venom production [7,17,18,19].

As previously reported for *Nephilingis cruentata* and *Stegodyphus mimosarum*, the astacin multigenic family is the most highly expressed and important proteolytic enzyme, followed by cathepsin L and the serine peptidase trypsin. Activities partially reflected the transcriptomic data, with astacin as the most active enzyme, followed by trypsin and cathepsin-L. Despite astacin showing the highest activity, there is wide dispersion in individual values across batches. These differences may be due to differences in the feeding states of the individuals used, with higher astacin activity expected in fasting specimens. This is based on proteomic data from Fuzita and co-authors [7], who found high concentrations of peptides and astacin activity in the MD specimens from fasting spiders and in their digestive fluid. These multiple astacin isoforms are also present in the proteomic data from the digestive fluid of *Stegodyphus minosarum*, but they are distinct from those in the digestive fluid of *Acanthoscurria geniculata*. Walter and co-authors [20] suggest that this could be related to the evolution of spiders, as Theraphosidae are more basal spiders. The most recent spider phylogenetic analysis based on mitochondrial genome sequencing [21,22] corroborates the proximity of the Uloboridae family to other orb-weaving spiders such as *Stegodyphus* sp., *Nephilingis* sp. and *Trichonephila* sp. The correlation of astacin isoform number and phylogeny is also supported by the low number of astacin genes in other basal Arachnids, such as scorpions [22]. The only protein that showed disparate results in terms of activity relative to transcript levels was cathepsin-L. As in other spiders, cathepsin-L is highly expressed at the MD in its zymogen form. After acidic activation, cathepsin-L exhibits high activity and is involved in both phases of digestion: extraoral and intracellular. The involvement of enzymes such as cathepsin-L in the digestive processes of *Z. geniculata* and *Uloborus* sp. confirms the presence of acidic peptidases in the digestive systems of spiders, which are responsible for prey liquefaction and intracellular digestion [6,7,23]. However, lower enzyme activity was observed in *Z. geniculata compared* to *Uloborus sp.* One hypothesis for this difference is a distinct processing of cathepsin-L zymogen activation. Another possibility is that the differences in cathepsin activity between these two species may be explained by differences in feeding habits (different prey types) and feeding status (fasted or fed individuals). 

Another peptidase family that is involved in protein degradation is the serine peptidases. In all spiders previously studied, S1 family enzymes were identified, both as contig sequences through transcriptomic analysis and as peptides through proteomics [23,24]. However, serine peptidase assays in MD homogenized samples from *Nephilengys cruentata*, *Loxosceles gaucho*, *Trichonephila clavipes*, *Phoneutria nigriventer*, and *Uloborus* sp. did not show measurable activity. *Z. geniculata* data, on the other hand, show high trypsin activity, compared to the absence of measurable activity in the digestive systems of the aforementioned spiders. This could indicate a serine peptidase with different specificity than in other spiders, or that the trypsin activity observed may originate from the prey, or even resemble venom-like serine peptidases from other spiders, which also contain only the catalytic domain.

Protein degradation also relies on the activities of exopeptidases, such as carboxypeptidase and aminopeptidase. Among them, carboxypeptidase is both the most expressed and the most active enzyme.

**Figure 5:**
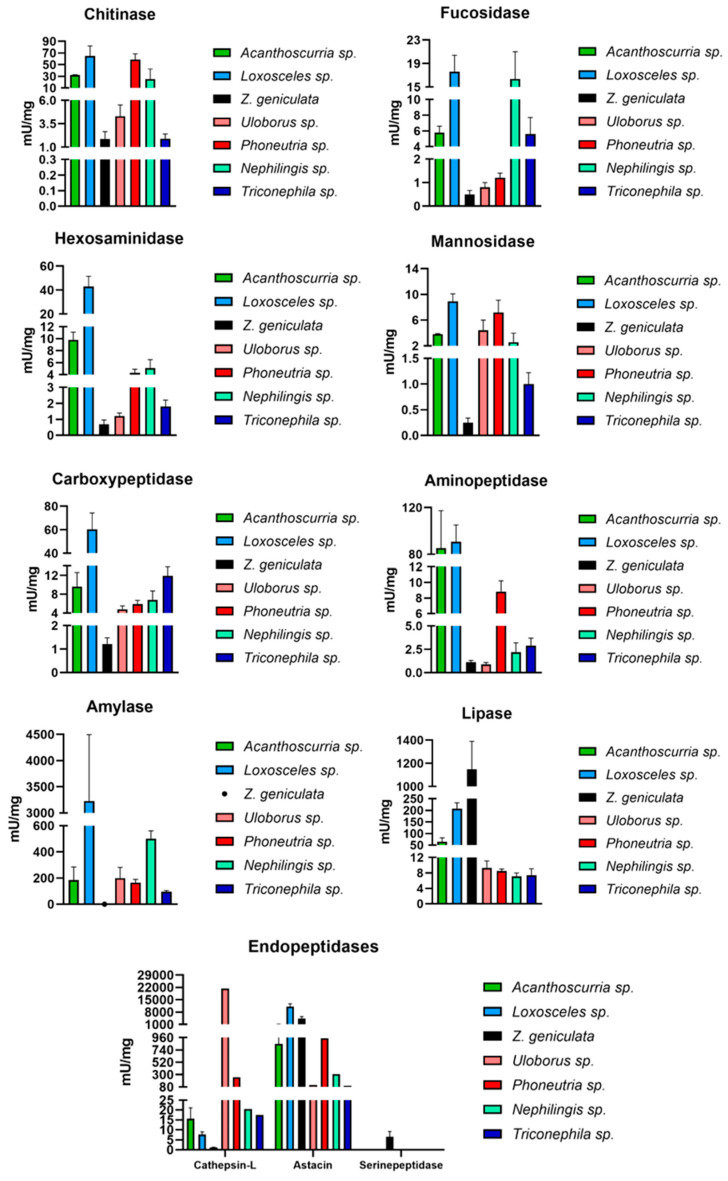
Comparison of specific activities (mU/mg) of the main enzymes involved in digestion in spiders (chitinase, fucosidase, hexosaminidase, mannosidase, carboxypeptidase, aminopeptidase, cathepsin-L, amylase, astacin and lipase) in distinct spider species. Black: *Zosis geniculata*; Blue: *Loxosceles* sp.; Pink: *Uloborus* sp.; Cyan green: *Nephilingis* sp.; Red: *Phoneutria* sp.; Navy blue: *Trichonephila* sp.; Dark green: *Acanthoscurria* sp. Spider genera were placed according to phylogeny from most basal to more derived ones. Graphs were generated in GraphPad.

Within the carbohydrases, chitinase is the most representative enzyme, likely because spiders’ common prey, such as arthropods, have a chitinous exoskeleton. Thus, the cuticular exoskeleton is the first barrier that must be degraded by enzymes from the digestive fluid and MD [7]. Chitinases are also the most abundant enzymes in *Nephilengys cruentata*, especially in fasting spiders [7]. In contrast, *Z. geniculata* shows lower chitinase activity compared to other arachnid species (Figure 5). However, there is no statistically significant difference in chitinase activity between *Z. geniculata* and *Uloborus* sp., suggesting this may represent a family-specific enzymatic trait. Additionally, mannosidase and fucosidase contribute to intracellular carbohydrate digestion, which may explain the observed activity levels of these enzyme classes (Figure 5). Amylase activity was not measurable in the experimental conditions. However, TPM values sum for amylase are around 400. There are a few possible explanations for this occurrence: 1- mRNA production and translation, as well as protein synthesis, occur at distinct periods during feeding conditions [7]. The asynchronousness of mRNA and protein synthesis has already been observed and discussed; 2 another possibility is the amylase stability. The MD homogenate sample is prepared in the absence of peptidase inhibitors, as these would serve as a source of various enzymes. Amylase might be a substrate for astacin, cysteine, and serine peptidase in the homogenate. Other sample-preparation protocols could be tested to measure starch degradation in *Z. geniculata* MD exclusively. However, in other spiders this phenomenon was not observed, mainly in Orbiculariae spiders like *Trichonephila* and *Nephilingis*, and even in *Uloborus sp* 3- *Z. geniculata*, which did not have feeding control, which could also interfere with the quantity of amylase activity.

The lipase was the only enzyme that showed higher activity in *Z. geniculata* compared to all other arachnids, with a significant difference particularly between *Z. geniculata* and *Uloborus* sp., which belong to the same family (Figure 5). In other comparisons among Uloboridae species, the high lipase activity may be mainly related to differences in feeding status among the different spider species used in the experiments. However, *Zosis* sp. has at least 7 distinct lipase transcripts, totaling a TPM of 2400, which is higher than usually observed in spiders.

The inhibition assays revealed the presence of bioactive molecules that modulate serine peptidase activity. Although the exact identity of the inhibitor was not determined, the extract from the *Z. geniculata* digestive system inhibited trypsin activity. When the fraction of the *Z. geniculata* homogenate was heated to 100 °C, the sample was enriched by removing its proteolytic enzymes, allowing the detection of thermostable inhibitors. These inhibitors are generally small molecules rich in disulfide bonds, which do not break in the absence of reducing agents, unlike ionic interactions [25]. Therefore, the extract maintained its inhibitory power even after boiling. The heated *Zosis* sp. extract was more effective at inhibiting insect trypsins (cricket and beetle) than bovine trypsin. Structural differences between insect and mammalian trypsins can explain this difference in inhibition. Insect trypsins generally have fewer disulfide bonds, which makes them more susceptible to inhibition by the compounds present in the extract. The stability of these inhibitors at high temperatures makes them promising for industrial and pharmaceutical applications, especially in formulations that need to withstand harsh conditions.

### 3.2. Identification and Functions of Toxin-like and Peptidase Inhibitors in the MD of Uloboridae Spiders

The transcriptome analysis performed in *Uloborus diversus* [14], *Uloborus plumipes* [15] and *Z. geniculata* set a stone on the presence of toxin-like proteins at MD and corroborated the literature data of the presence of peptidase inhibitors at the digestive tract of Arachnida species, including spiders. According to Peng and co-authors [15], spiders of the Uloboridae family may have undergone a phenomenon similar to natural selection in the use of venom. In this case, as venom ceased to be essential to the survival of these spiders, natural selection may have favored the loss of venom glands. In the location where venom glands are found in araneomorph spiders, only muscle bundles are present in Uloboridae individuals [15]. The main hypothesis is that uloborids have replaced venom with toxic molecules in their digestive fluids, as these fluids appear to be lethal to prey, as confirmed by Weng [26]. The presence of toxins in the digestive system of *Z. geniculata* was also confirmed by transcriptomic data with high identity to the toxins described for *Uloborus plumipes* [15]. 

Among different toxins and inhibitors present in the MD transcriptome of *Z. geniculata*, diverse sequences corresponding to the atracotoxin family were identified. These toxins act on neuronal ion channels and are found in the venom of *Atrax robustus* spiders [27]. Expression values of these sequences vary, with the highest reaching 10,795 TPM. The presence of atracotoxins was also observed for *Uloborus plumipes* and even in venomous spiders such as *Nephilengys cruentata*, *Acanthoscurria geniculata*, and *Stegodyphus mimosarum*, where toxins and inhibitors were identified in the digestive fluid and MD. Although the primary reported function of atracotoxins is their neurotoxic effect, the isolated atracotoxin NcTI from the MD of *Nephilingis cruentata* exhibited trypsin inhibitory activity [8]. In terms of bioprospecting, seven atracotoxin sequences were identified in the *Z. geniculata* transcriptome. This is only one example of the several possible molecules present in spiders, which could have biotechnological interest and reinforce spiders as new sources for identifying novel inhibitors and bioactive molecules. The presence of toxins at the MD of Uloboridae spiders might lead us to two distinct hypothesis: (1) that digestive toxins may play a role during prey capture and death in Uloboridae spiders, as prey degradation and death processes begin only after the digestive fluids are deposited and as suggested by Peng et al. [15], has indeed integrated toxins into their digestive system, primarily to assist in prey immobilization or (2) that digestive tract has the original venom-like toxin genes involved in the digestive process and that due to gene duplication and differentiation along evolution has become more specialized in toxin functions. This hypothesis is reinforced by the presence of toxin-like proteins in the digestive tracts of distinct spider families, and by recent reports of peptides with paralyzing activity found in the digestive systems of venomless arachnids such as mites and ticks. Another aspect that corroborates the second hypothesis is the phylogenetic analysis of phospholipase D [14] showing that *Uloborus* sp. has a digestive system rich in hydrolytic enzymes as well as other spiders, ticks, and even the horseshoe crab, which is a basal representative of Chelicerata. Valladão et al. and Pedroso et al. [4,28] also demonstrated the distinction between specificity and mutation in the *venom of the Loxosceles* genus, which is highly toxic, a distinction specific to this genus of spiders. It is further proposed that the presence of redundant toxins may serve as an evolutionary compensation system, ensuring prey immobilization when venom alone is insufficient. In larger species such as *A. geniculata*, which possess robust chelicerae capable of killing prey through mechanical trauma, the amount of toxins in the digestive fluid tends to be lower. In such cases, physical strength is the primary resource [15]. In contrast, smaller spiders like *Z. geniculata* may benefit from a combination of strategies. In Figure 4, the structures of the most highly expressed sequences (highest TPM value) from the five prominent families of peptidase inhibitors found in the transcriptome of *Z. geniculata* are presented: Kunitz, TIL, Cystatin, Kazal, Serpins, and Atracotoxins. The most expressed protein containing a Kunitz domain in *Z. geniculata* is annotated as a papilin-like. BlastP of this papilin against Arachnida sequences evidenced the presence of 1690 proteins (e-value 1 × 10^−10^ and at least 50% of query cover).

A total of 622 belong to spider species, 994 to mites and ticks, and 56 to scorpions. Between spiders, Orbiculariae spiders produce 563 papillins. Similar to the multigenic families of metallopeptidases, which are larger in spiders [23], there appears to be an increase in the presence of Kunitz domains in Orbiculariae spiders, including Uloboridae. This enhancement can be related to spider prey using distinct capture strategies, including flying insects such as moths and butterflies. The increase in the presence of inhibitory molecules in Orbiculariae could reflect a coevolutionary process between Orbiculariae spiders and Lepidoptera similar to that observed between plants and Lepidoptera species [29,30,31,32]. The same seems true for mites and ticks, as ticks presented larger Kunitz family representatives (in medium per species = 46) than mites (in medium per species = 3.3), possibly due to their blood-feeding habits. Another family of inhibitors that is mechanically diverse from Kunitz, TIL and Kazal inhibitors are the serpins [25,26]. Serpins are proteins with a molecular mass of 60–70 kDa that belong to a superfamily of serine peptidase inhibitors, sharing a common evolutionary origin and exhibiting highly homologous structural sequences.

Thus, the exploration of *Z. geniculata*’s digestive system contributes to the evolutionary understanding of inhibitory and toxicity mechanisms in non-venomous spiders.

## 4. Materials and Methods

### 4.1. Sample Preparation and Protein Quantity Estimation

Adult females and males of the species *Z. geniculata* (Figure 1) were collected (−23.5474078, −46.6926179) and immediately transported to the Laboratory of Biochemistry at Instituto Butantan. There was no control over the feeding status of these spiders (fed or fasted), nor was the number of males and females distinguished.

The spiders were immobilized on ice for approximately 5 to 10 min. The spiders were dissected under a stereomicroscope to isolate the MD in cold isotonic saline (NaCl 342 mM) using tweezers. Samples containing four MD were homogenized in 1.0 mL of iced ultrafiltered water in a Potter-Elvehjem homogenizer. The samples were centrifuged at 16,000× *g* at 4 °C for 30 min to obtain the soluble fraction, which were used as enzyme source for the assays. Fifteen different MD homogenate samples of *Z. geniculata* were used for enzymatic and inhibitory assays. At the end of the preparation, the samples were aliquoted and stored at −20 °C until use.

After preparation of the homogenates, protein quantification was estimated using the bicinchoninic acid (BCA) method [33], a standard curve was prepared with different concentrations of egg albumin from a stock solution (0.2 mg/mL). The standard curve and the sample reactions were incubated at 60 °C for 30 min. The absorbance was measured in a SpectraMax 190 spectrophotometer (Molecular Devices, San Jose, CA, USA)—at a wavelength of 562 nm.

### 4.2. Enzymatic Assays

Enzyme assays, as previously described, were performed with the soluble fraction of μa homogenate sample containing four *Z. geniculata* adult MD. In total, 15 distinct homogenate samples were analyzed. This study has measured 11 distinct enzymes, categorized into three primary classes: carbohydrases (α-amylase, hexosaminidase, α-L-fucosidase, α-mannosidase, and chitinase), peptidases (serine peptidase, cysteine peptidase, aminopeptidase, carboxypeptidase, and metallopeptidase) and, lipase [7,23]. 

The main digestive enzyme activities of *Z. geniculata* were assayed in 96-well plates, with appropriate times for each enzyme, as well as the pH, buffer solutions, and substrate conditions. Enzyme and substrate negative controls were used. The activities were measured using fluorescence detected by the SpectraMax Gemini XPS spectrofluorimeter (Molecular Devices) or absorbance at Spectramax 190 spectrophotometer. All enzyme activity was measured in U (µmol of product per minute), except astacin, which was calculated as fluorescence units per minute. At the end of the enzymatic assays, statistical analysis of the data was performed using GraphPad Prism software version 10.0.0 for Windows [34]. The nonparametric Kruskal–Wallis test and analysis of variance (ANOVA) were performed at α = 0.05, with the median as the measure of central tendency [34]. The same software was used to visualize the results through scatterplots.

### 4.3. Inhibition Assays

Inhibition assays were performed to observe and quantify the action of inhibitor sources on trypsin. Inhibition assays were carried out to determine whether the MD of *Z. geniculata* homogenate could inhibit trypsin activity (bovine, *Gryllus* sp., and *Zophobas morio*), considering that these samples have previously described trypsin activities [20], using N-benzoyl-D-L-arginine-p-nitroanilide as trypsin substrate. 

For these inhibition assays, we used MD soluble fraction homogenates from *Z. geniculata* as the inhibitor source, and isolated midgut homogenate samples from *Gryllus* sp. (1 midgut/homogenate) and *Zophobas morio* (5 MD/homogenate) as the enzyme source. We subjected the MD soluble fraction sample from *Z. geniculata* to two different conditions: heat-treated (100 °C) and non-heat-treated. The heat-treated samples were boiled for 20 min at 100 °C and then centrifuged for 10 min at 16,000× *g* and 4 °C, while the other samples were used immediately after thawing. The heat treatment was used to observe the presence of heat-resistant inhibitors in the *Z. geniculata* homogenate, as these inhibitors maintain their structure and function even at high temperatures [35], and to avoid the presence of *Z. geniculata* trypsin activity.

The purified bovine trypsin (Merck-T1426) sample used was diluted 14-fold from the stock solution 50 µg/µL. The *Gryllus* sp. sample and the *Z. morio* sample were diluted 10- and 20-fold, respectively, twice in 0.1 M Tris-HCl buffer, pH 8.0, containing 20 mM CaCl_2_. The volumes of the *Z. geniculata* samples used were 2, 4, 6, 8, and 10 µL. Samples of *Z. geniculata*, bovine trypsin, and the different trypsin sources (cricket and beetle) were placed on a transparent 96-well plate, along with the appropriate buffer, in a 1:1 proportion. The plate was incubated for 20 min in a Spectramax 190 spectrophotometer at 37 °C. After this incubation time, the substrate 1.0 mM α-N-Benzoyl-D-L-Arginine-p-nitroanilide diluted in 0.1 M Tris-HCl buffer was added. After adding the substrate, the plate was returned to the spectrophotometer for kinetic readings at 410 nm for 30 min, with readings every 10 min, at the same temperature as before. After obtaining the data, initial velocities were calculated for each assay, and the percentage inhibition of trypsin activity in cricket, beetle, and bovine trypsins by the *Z. geniculata* sample was calculated by quantifying the remaining activity in comparison to the enzyme control. These tests were performed with distinct seven biological replicates.

For statistical analysis, GraphPad Prism software version 10.0.0 for Windows was used again, using the nonparametric Kruskal–Wallis test (median) and ANOVA (α = 0.05) [34]. For testing significance between groups, Tukey’s multiple comparisons test was used, with *p*-values < 0.05. The same software was used to visualize the scatter plots. A positive control with buffer, trypsin source, and substrate was also performed, as was a negative control with buffer and substrate, for a final concentration of 150 µL in each well. They were subjected to the same treatment as the other samples.

### 4.4. RNA Extraction, Sequencing Data Analysis and Molecular Modeling

RNA extraction was performed to obtain total RNA for cDNA sequencing on an Illumina NextSeq to generate the MD transcriptome of *Z. geniculata*. A pre-established protocol with TRIzol was followed according to the manufacturer’s instructions (Invitrogen, Waltham, MA, USA) using three batches (3 MD/batch) of *Z. geniculata* MD. The extracted RNA was resuspended in 20 µL of water containing diethyl pyrocarbonate (DEPC) and stored at −80 °C. After extraction, the samples were sent to NGS Genomic Solutions (ngsgenomica.com.br) for cDNA library assembly and PE sequencing at 2 × 150 base pairs, approximately 15,000,000 clusters each, on the NextSeq Illumina platform. Qualitative RNA analysis was performed using a Nanodrop spectrophotometer (Thermo Fisher, Waltham, MA USA). The quality of the reads was assessed using the FastQC report [36], followed by trimming using Trimmomatic [37]. Ribosomal RNA removal was performed using Bbduk (Joint Genome Institute) using reference sequences from the ribokmers.fa.gz file. Contigs were assembled from the reads using Trinity v2.15.1 [24,37]. ORF prediction was performed using TransDecoder [38]. HMMER Search, which accesses the Pfam database, was also used for domain prediction [39]. Only the longest isoforms obtained with the Trinity script get_longest_isoform_seq_per_trinity_gene.pl were retained in the set of annotated proteins. Functional annotation of the contigs was performed with Diamond and BlastP, using the RefSeq invertebrate protein database (ftp.ncbi.nlm.nih.gov/refseq/release/invertebrate/, 20 September 2024) as reference sequences [40]. In addition, InterProScan was used for domain prediction. The completeness of the assembly was evaluated by searching for single-copy genes in Eukaryota and Arachnida using BUSCO [41]. RSEM with Bowtie2 were used to map the reads and estimate abundance [42]. Expression values were normalized and reported as TPM (Transcripts Per Million) and the mean between the three samples was used as the TPM values.

OmicsBox v3.3.2 [17] was used to integrate and interpret the data obtained during the annotation step. In addition to incorporating the data and facilitating interpretation, the software also displayed the distribution of annotated sequences among different arthropod species. Graphs were generated using GraphPad Prism version 10.0.0 for Windows. To model the most highly expressed sequences (with the highest TPM values) from the main families of peptidase inhibitors (serpins, atracotoxins, Kunitz, Kazal-type, Cystatin, and TIL), AlphaFold3 was used.

## 5. Conclusions

This study presents the first integrated biochemical and transcriptomic characterization of the digestive system of the non-venomous spider *Z. geniculata*, revealing it as an unexplored source of peptidase inhibitors with significant biotechnological potential due to the diversity of inhibitor families and thermal stability. The enzymatic profile indicated a high hydrolytic capacity, with elevated activities of lipases and peptidases (astacin, cathepsin L and trypsin), supporting the hypothesis that Uloboridae compensate for the evolutionary loss of venom glands through a highly active digestive fluid. Transcriptomic analysis revealed a diverse repertoire of inhibitor families—including Serpin, Kunitz, Cystatin, Kazal, TIL, and atracotoxins. Taken together, these findings expand current knowledge of Uloboridae digestive physiology and support the notion that non-venomous spiders harbor a molecular arsenal comparable in complexity to that of venomous species.

## Figures and Tables

**Table 1:** Specific activity of digestive enzymes in the soluble fraction of *Zosis geniculata* MD homogenates.

Enzyme	Specific Activity (mU·mg^−1^)
**Carbohydrases**	
chitinase	1.88 ± 0.77 mU·mg^−1^
hexosaminidase	0.69 ± 0.27 mU·mg^−1^
α-L-fucosidase	0.50 ± 0.17 mU·mg^−1^
α-mannosidase	0.25 ± 0.09 mU·mg^−1^
α-Amylase	non-activity
**Peptidases**	
carboxypeptidase	1.22 ± 0.26
aminopeptidase	1.12 ± 0.19
astacin	±
trypsin	6.61 ± 2.51
cathepsin-L	1.14 ± 0.17
**Lipase**	1150.3 ± 239.9

**Table 2:** Expression values (TPM > 50) of digestive enzymes identified in the digestive system transcriptome of *Z. geniculata*.

Enzymes	Highest TPM	Contigs Numbers	Individuals TPMs	Sum of TPMs
Chitinase	7869.05	4	7869.05; 4963.17; 63.56; 61.93	12,957.70
Hexosaminidase	241.69	2	241.69; 168.56	410.26
α-L-Fucosidase	125.37	1	125.37	125.37
α-Mannosidase	60.20	2	60.20; 56.07	116.27
α-Amylase	388.72	3	9.24; 6.02; 388.72	403.97
Carboxypeptidase	1090.72	4	1090.72; 156.63; 63.0; 55.23	1365.57
Aminopeptidase	260.45	4	260.45; 173.70; 90.44; 56.76	581.35
Astacin	10,562.63	17	10,562.63; 4437.86; 2830.45; 2381.76; 1700.29; 1567.28; 1379.01; 1017.14;	28,534.22
			551.66; 440.52; 427.30; 339.43; 296.82; 232.64; 173.34; 107.35; 88.75	
Trypsin	108.20	3	108.20; 37.31; 27.76	173.27
Cathepsin L	3873.51	6	3873.51; 2310.79; 1457.78; 923.40; 733.21; 163.63	9462.30
Lipase	779.42	6	779.42; 495.47; 369.42; 249.88; 237.87; 95.52	2227.58

The table shows enzymes with Transcripts Per Million (TPM) values greater than 50, identified in the midgut transcriptome. Information includes enzyme type, gene ID, TPM value, functional annotation, and the corresponding enzyme family, based on bioinformatic analyses and comparisons with functional databases. The highest TPM value for the amylase enzyme corresponds to the contig identified as a low-quality protein.

**Table 3:** Toxin sequences found in the digestive system transcriptome of *Zosis geniculata* and their expression values.

Sequence/ID	Sequence Description	Pfam Domains	TPM
DN1235_c0_g1_i1.p1	U24-ctenitoxin-Pn1a-like	Thyroglobulin_1	20,066.44
DN1146_c0_g1_i5.p1	U24-ctenitoxin-Pn1a-like	Thyroglobulin_1	11,540.43
DN746_c0_g1_i1.p1	U1-hexatoxin-Iw1e-like	MIT_LIKE_ACTX, Prokineticin	10,795.41
DN1489_c0_g1_i1.p1	astacin-like metalloprotease toxin 5	Astacin	10,562.63
DN2079_c0_g1_i1.p1	U24-ctenitoxin-Pn1a-like isoform X1	Thyroglobulin_1	9593.79
DN4294_c0_g1_i2.p1	U21-ctenitoxin-Pn1a-like isoform X1	Trypsin	9575.33
DN626_c0_g1_i1.p1	U24-ctenitoxin-Pn1a-like	Thyroglobulin_1	5606.36
DN6417_c0_g1_i1.p1	astacin-like metalloprotease toxin 5	Astacin	4437.86
DN34549_c0_g1_i1.p1	astacin-like metalloprotease toxin 5	Astacin	2830.45
DN1039_c0_g1_i1.p1	U24-ctenitoxin-Pn1a-like	Thyroglobulin_1	2454.28
DN214_c0_g1_i2.p1	astacin-like metalloprotease toxin 5	Astacin	2381.76
DN72_c0_g1_i1.p1	astacin-like metalloprotease toxin 5	Astacin	1700.29
DN982_c0_g1_i16.p1	astacin-like metalloprotease toxin 5	Astacin	1567.28
DN1389_c0_g1_i1.p1	U3-aranetoxin-Ce1a-like	MIT_LIKE_ACTX	1414.86
DN4141_c0_g1_i3.p1	astacin-like metalloprotease toxin 1	Astacin, MAM	1379.01
DN2163_c0_g1_i1.p1	toxin CSTX-20-like	Prokineticin	1212.46
DN5_c0_g1_i15.p1	astacin-like metalloprotease toxin 5	Astacin	1017.14
DN2267_c0_g1_i1.p1	U9-ctenitoxin-Pr1a-like	MIT_LIKE_ACTX	862.07
DN1042_c0_g1_i1.p1	astacin-like metalloprotease toxin 5	Astacin	551.66
DN1222_c0_g1_i1.p1	astacin-like metalloprotease toxin 5	Astacin, Peptidase_M10	440.52
DN1261_c0_g1_i1.p1	astacin-like metalloprotease toxin 5	Astacin	427.30
DN33_c0_g1_i7.p1	U33-theraphotoxin-Cg1c	Prokineticin	392.00
DN3333_c0_g1_i2.p1	U3-aranetoxin-Ce1a-like	MIT_LIKE_ACTX	385.53
DN1_c0_g1_i2.p1	astacin-like metalloprotease toxin 1	Astacin	339.43
DN762_c0_g1_i1.p1	astacin-like metalloprotease toxin 5	Astacin	296.82
DN872_c0_g1_i1.p1	U9-ctenitoxin-Pr1a-like	MIT_LIKE_ACTX, Prokineticin	286.02
DN470_c0_g1_i1.p1	astacin-like metalloprotease toxin 1	Astacin	232.64
DN650_c0_g1_i1.p2	U3-aranetoxin-Ce1a-like	MIT_LIKE_ACTX	228.77
DN541_c0_g1_i1.p1	astacin-like metalloprotease toxin 5	Astacin, Peptidase_M10	173.34
DN387_c0_g1_i1.p1	U24-ctenitoxin-Pn1a-like	Thyroglobulin_1	152.62
DN672_c0_g1_i1.p1	U21-ctenitoxin-Pn1a-like	Trypsin, Trypsin_2	108.07
DN793_c0_g1_i3.p1	astacin-like metalloprotease toxin 5	Astacin	107.35
DN4977_c0_g1_i1.p1	dermonecrotic toxin StSicTox-betaIB1i-like	GDPD_2, GDPD	90.92
DN4422_c0_g1_i1.p1	astacin-like metalloprotease toxin 5	Astacin	88.75
DN877_c0_g1_i2.p1	scoloptoxin SSD14-like isoform X1	G_glu_transpept	72.03
DN6557_c0_g1_i1.p1	U3-aranetoxin-Ce1a-like	-	61.93
DN2620_c0_g1_i1.p1	U24-ctenitoxin-Pn1a-like	Thyroglobulin_1	56.67
DN1186_c0_g1_i4.p1	U21-ctenitoxin-Pn1a-like	Trypsin	53.39
DN1235_c0_g1_i1.p1	U24-ctenitoxin-Pn1a-like	Thyroglobulin_1	20,066.44
DN1146_c0_g1_i5.p1	U24-ctenitoxin-Pn1a-like	Thyroglobulin_1	11,540.43
DN746_c0_g1_i1.p1	U1-hexatoxin-Iw1e-like	MIT_LIKE_ACTX, Prokineticin	10,795.41
DN1489_c0_g1_i1.p1	astacin-like metalloprotease toxin 5	Astacin	10,562.63
DN2079_c0_g1_i1.p1	U24-ctenitoxin-Pn1a-like isoform X1	Thyroglobulin_1	9593.79
DN4294_c0_g1_i2.p1	U21-ctenitoxin-Pn1a-like isoform X1	Trypsin	9575.33
DN626_c0_g1_i1.p1	U24-ctenitoxin-Pn1a-like	Thyroglobulin_1	5606.36
DN6417_c0_g1_i1.p1	astacin-like metalloprotease toxin 5	Astacin	4437.86
DN34549_c0_g1_i1.p1	astacin-like metalloprotease toxin 5	Astacin	2830.45
DN1039_c0_g1_i1.p1	U24-ctenitoxin-Pn1a-like	Thyroglobulin_1	2454.28
DN214_c0_g1_i2.p1	astacin-like metalloprotease toxin 5	Astacin	2381.76
DN72_c0_g1_i1.p1	astacin-like metalloprotease toxin 5	Astacin	1700.29
DN982_c0_g1_i16.p1	astacin-like metalloprotease toxin 5	Astacin	1567.28
DN1389_c0_g1_i1.p1	U3-aranetoxin-Ce1a-like	MIT_LIKE_ACTX	1414.86
DN4141_c0_g1_i3.p1	astacin-like metalloprotease toxin 1	Astacin, MAM	1379.01
DN2163_c0_g1_i1.p1	toxin CSTX-20-like	Prokineticin	1212.46
DN5_c0_g1_i15.p1	astacin-like metalloprotease toxin 5	Astacin	1017.14
DN2267_c0_g1_i1.p1	U9-ctenitoxin-Pr1a-like	MIT_LIKE_ACTX	862.07
DN1042_c0_g1_i1.p1	astacin-like metalloprotease toxin 5	Astacin	551.66
DN1222_c0_g1_i1.p1	astacin-like metalloprotease toxin 5	Astacin, Peptidase_M10	440.52
DN1261_c0_g1_i1.p1	astacin-like metalloprotease toxin 5	Astacin	427.30
DN33_c0_g1_i7.p1	U33-theraphotoxin-Cg1c	Prokineticin	392.00
DN3333_c0_g1_i2.p1	U3-aranetoxin-Ce1a-like	MIT_LIKE_ACTX	385.53
DN1_c0_g1_i2.p1	astacin-like metalloprotease toxin 1	Astacin	339.43
DN762_c0_g1_i1.p1	astacin-like metalloprotease toxin 5	Astacin	296.82
DN872_c0_g1_i1.p1	U9-ctenitoxin-Pr1a-like	MIT_LIKE_ACTX, Prokineticin	286.02
DN470_c0_g1_i1.p1	astacin-like metalloprotease toxin 1	Astacin	232.64
DN650_c0_g1_i1.p2	U3-aranetoxin-Ce1a-like	MIT_LIKE_ACTX	228.77
DN541_c0_g1_i1.p1	astacin-like metalloprotease toxin 5	Astacin, Peptidase_M10	173.34
DN387_c0_g1_i1.p1	U24-ctenitoxin-Pn1a-like	Thyroglobulin_1	152.62
DN672_c0_g1_i1.p1	U21-ctenitoxin-Pn1a-like	Trypsin, Trypsin_2	108.07
DN793_c0_g1_i3.p1	astacin-like metalloprotease toxin 5	Astacin	107.35
DN4977_c0_g1_i1.p1	dermonecrotic toxin StSicTox-betaIB1i-like	GDPD_2, GDPD	90.92
DN4422_c0_g1_i1.p1	astacin-like metalloprotease toxin 5	Astacin	88.75
DN877_c0_g1_i2.p1	scoloptoxin SSD14-like isoform X1	G_glu_transpept	72.03
DN6557_c0_g1_i1.p1	U3-aranetoxin-Ce1a-like	-	61.93
DN2620_c0_g1_i1.p1	U24-ctenitoxin-Pn1a-like	Thyroglobulin_1	56.67
DN1186_c0_g1_i4.p1	U21-ctenitoxin-Pn1a-like	Trypsin	53.39

This table presents toxin sequences with Transcripts Per Million (TPM) values greater than 50, identified in the midgut transcriptome of *Z. geniculata*. Listed information includes toxin type, sequence ID, TPM value, and functional annotation based on similarity analyses with specialized databases. These data suggest the possible involvement of such toxins in the digestive system of the species, despite the absence of venom glands.

**Table 4:** Peptidase inhibitors found in the gut transcriptome of *Zosis geniculata* (TPM > 50).

Sequence/ID	Sequence Description	Pfam Domain	TPM
DN808_c0_g1_i17.p1	papilin-like	Kunitz_BPTI, TIL	20,275.43
DN485_c0_g1_i14.p1	L-cystatin-like	Cystatin, SQAPI	17,898.91
DN1157_c0_g1_i1.p1	nucleoprotein TPR-like	Apolipoprotein, YtxH, DUF6674, ApoLp-III, ApoC-I, DUF6415, DUF1664, Phasin, Rrn6_HB, OmpH, ATG17_like, ERp29, Gp-FAR-1, pAdhesive_17, Muted, phiKZ_IP, DUF5917, PGM_PMM_II, NleF_casp_inhib, PMC2NT, DUF6781, DUF2884, Exonuc_VII_L	12,144.23
DN882_c1_g1_i2.p1	probable chitinase 10 isoform X1	Glyco_hydro_18, CBM_14, Kunitz_BPTI	7869.05
DN458_c0_g1_i1.p1	uncharacterized protein LOC107445376	amfpi-1	6482.08
DN3530_c0_g1_i1.p1	actinia tenebrosa protease inhibitors-like	Kunitz_BPTI	3006.50
DN474_c0_g1_i1.p1	chymotrypsin inhibitor-like	TIL	2709.93
DN722_c0_g1_i2.p1	uncharacterized protein LOC129963689	amfpi-1	768.82
DN211_c0_g1_i3.p1	uncharacterized protein LOC129226369	Serpin	642.19
DN19_c0_g1_i3.p1	nascent polypeptide-associated complex subunit alpha, muscle-specific form-like isoform X1	Kunitz_BPTI	329.14
DN3168_c0_g1_i1.p1	insulin-like growth factor-binding protein-related protein 1 isoform X1	I-set, Ig_3, Ig_2, IGFBP, ig, Kazal_1, Kazal_2	220.72
DN232_c0_g2_i1.p1	low-density lipoprotein receptor-related protein 2-like	Ldl_recept_a, Ldl_recept_b, FXa_inhibition, cEGF, EGF_CA, DUF5050, SGL	216.94
DN4190_c0_g1_i2.p1	BPTI/Kunitz domain-containing protein-like isoform X1	Kunitz_BPTI	128.22
DN7140_c0_g1_i2.p1	SCO-spondin-like isoform X1	TIL	124.59
DN1698_c0_g1_i1.p1	serpin B6-like	Serpin	95.71
DN531_c0_g1_i1.p1	insulin-like growth factor-binding protein-related protein 1, partial	Ig_3, I-set, IGFBP, Kazal_2, Kazal_1, ig	70.66
DN908_c0_g1_i2.p1	low-density lipoprotein receptor-related protein 2-like	FXa_inhibition, Ldl_recept_b, SGL, Arylesterase	67.07
DN1501_c0_g1_i2.p1	fibulin-1-like	FXa_inhibition, cEGF, EGF_CA, Sushi, EGF, HYR, EGF_3	66.55
DN232_c0_g1_i1.p1	low-density lipoprotein receptor-related protein 2-like	Ldl_recept_b, FXa_inhibition, cEGF, EGF_CA, DUF5050, SGL	55.50
DN3114_c0_g1_i1.p1	SCO-spondin-like	TIL	52.16

The table presents the protein-coding sequences, their functional descriptions, the protein domains identified through Pfam analysis, and expression levels in TPM (Transcripts Per Million). The domains represent known peptidase inhibitor families, such as Kunitz_BPTI, TIL, Serpin, Cystatin, and Kazal, indicating potential roles as regulators of proteolytic activity in the spider’s digestive system. Uncharacterized or partially annotated sequences are also included to highlight candidate proteins for novel inhibitors.

## Data Availability

The raw RNA-seq sequence data from the *Zosis geniculata* midgut samples are available in the Short Read Archive (SRA) GenBank database https://www.ncbi.nlm.nih.gov/genbank: BioProject (PRJNA1286793), https://www.ncbi.nlm.nih.gov/genbank: BioSample (SAMN49798262, SAMN49798263, and SAMN49798264).

## Supplementary Materials

The following supporting information can be downloaded at: https://www.mdpi.com/article/10.3390/ijms27010186/s1.
