# Supplementary material for: Exploring Spiders Without Venom as New Sources of Peptidase Inhibitors
Source: Int J Mol Sci. 2025 Dec 24;27(1):186. doi: 10.3390/ijms27010186 (PMC12785369; doi:10.3390/ijms27010186)
Supplement: Supplementary file 1 [file ijms-27-00186-s001.zip › ijms-3939348-supplementary.pdf]

MolProbity Ramachandran analysis

kunitz_dominio_1_fold_2025_11_20_14_53_model_0.pdb, model 1

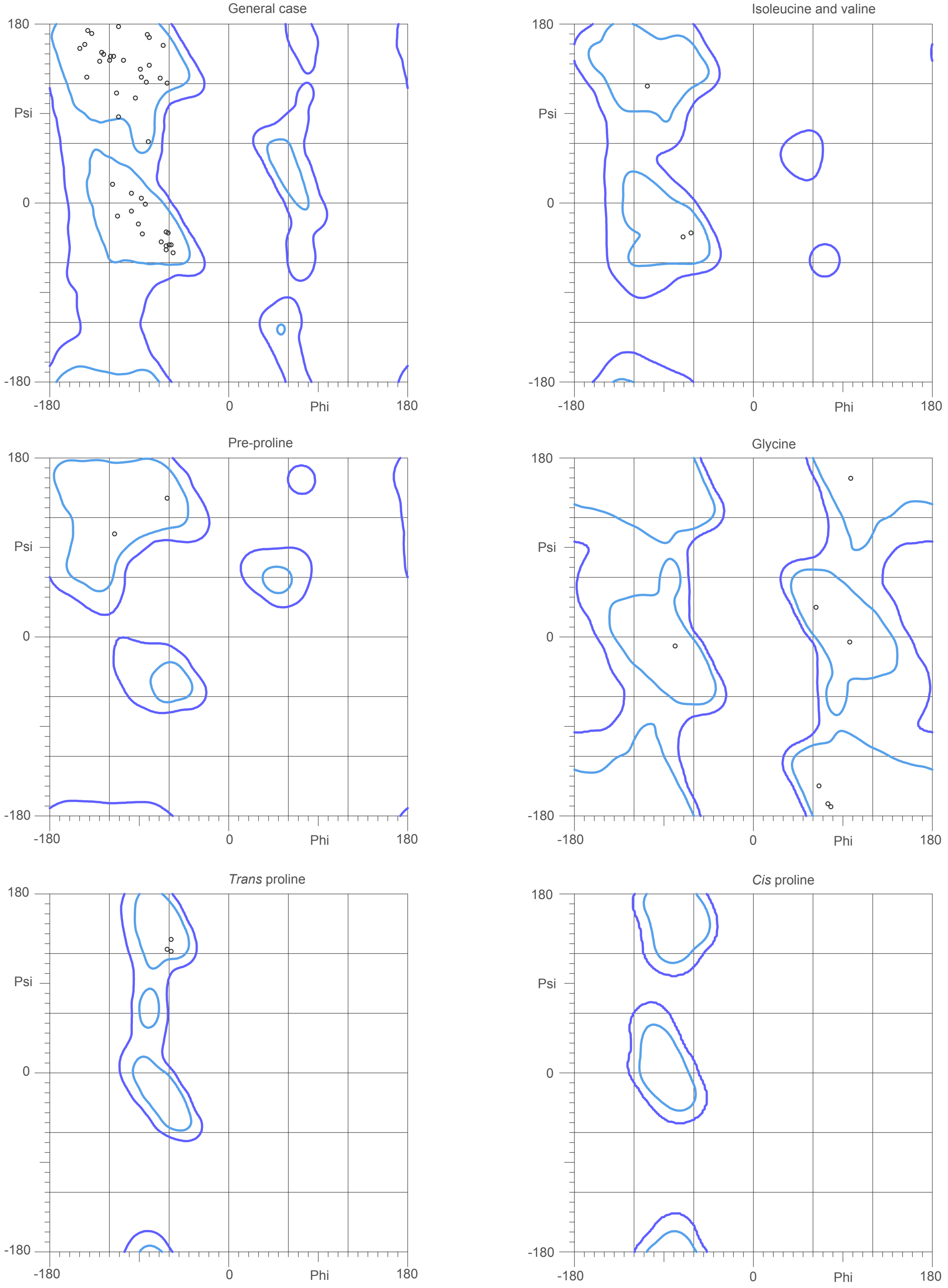

100.0% (57/57) of all residues were in favored (98%) regions.
100.0% (57/57) of all residues were in allowed (>99.8%) regions.

There were no outliers.

MolProbity Ramachandran analysis

cistatina_fold_2025_11_18_13_43_model_0.pdb, model 1

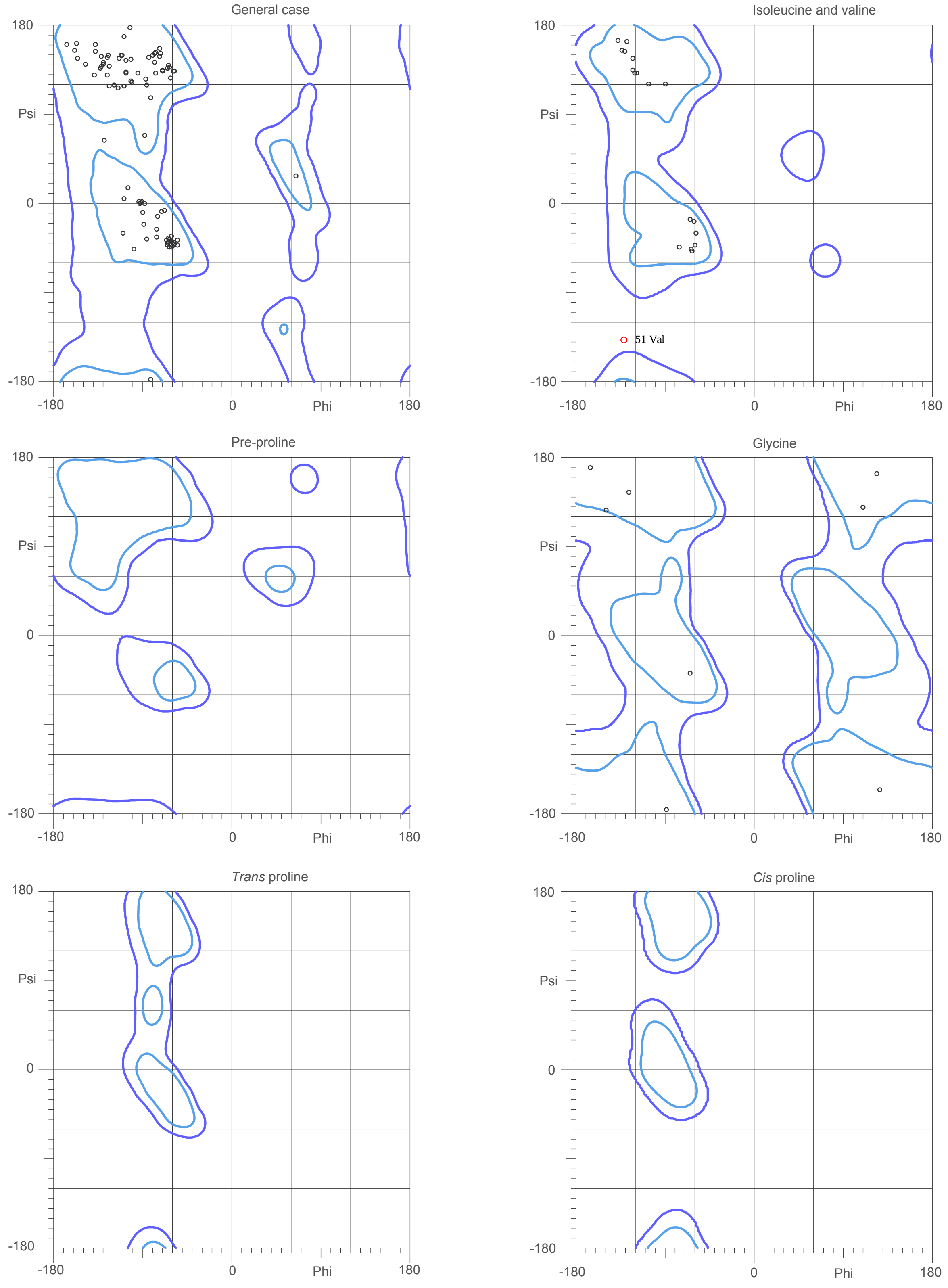

98.3% (117/119) of all residues were in favored (98%) regions.
99.2% (118/119) of all residues were in allowed (>99.8%) regions.

There were 1 outliers (phi, psi):
51 Val (-132.8, -138.8)

MolProbity Ramachandran analysis

TIL_fold_2025_11_18_14_22_model_0.pdb, model 1

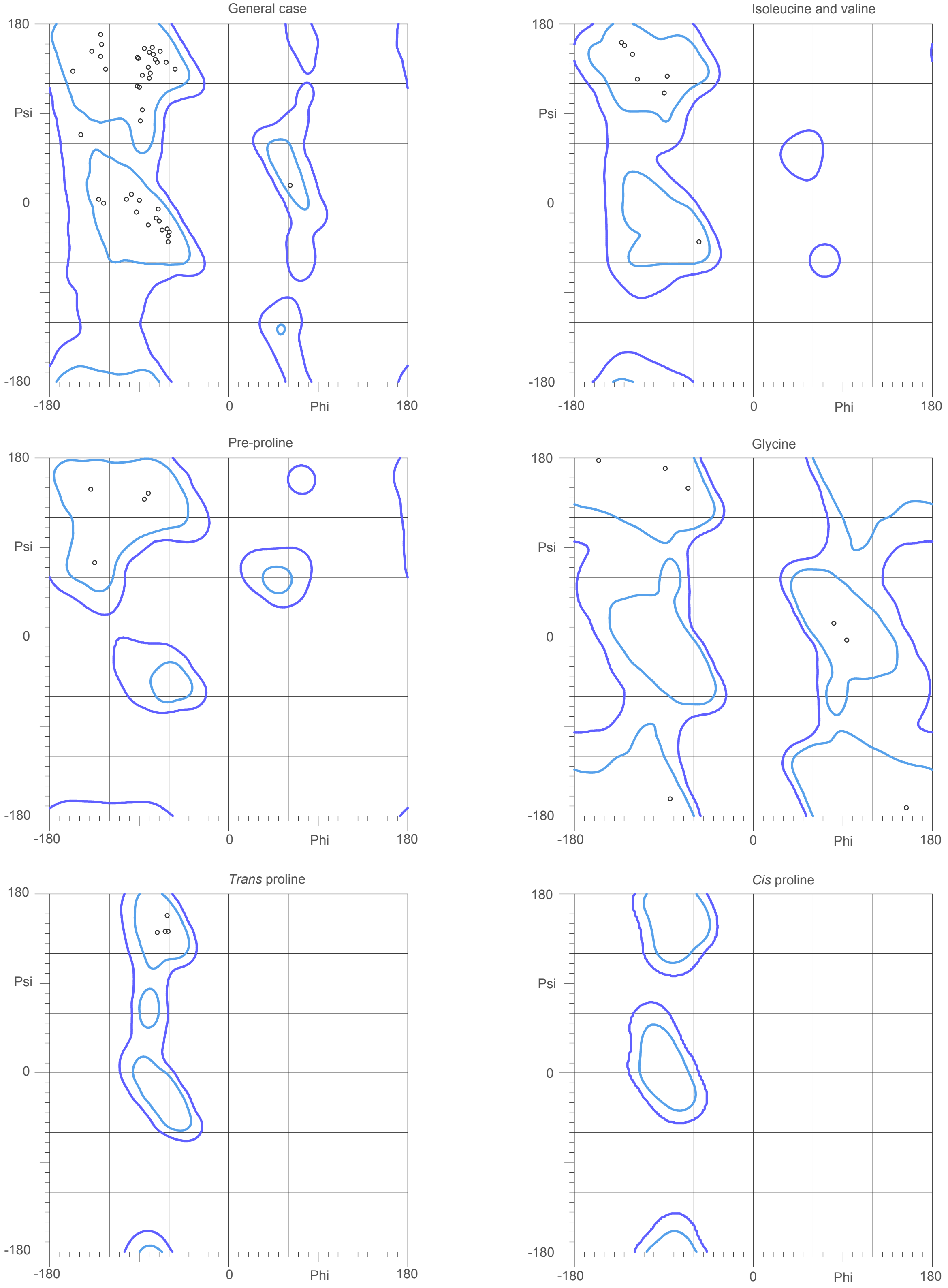

98.4% (63/64) of all residues were in favored (98%) regions.
100.0% (64/64) of all residues were in allowed (>99.8%) regions.

There were no outliers.

MolProbity Ramachandran analysis

cistatina_fold_2025_11_18_13_43_model_0.pdb, model 1

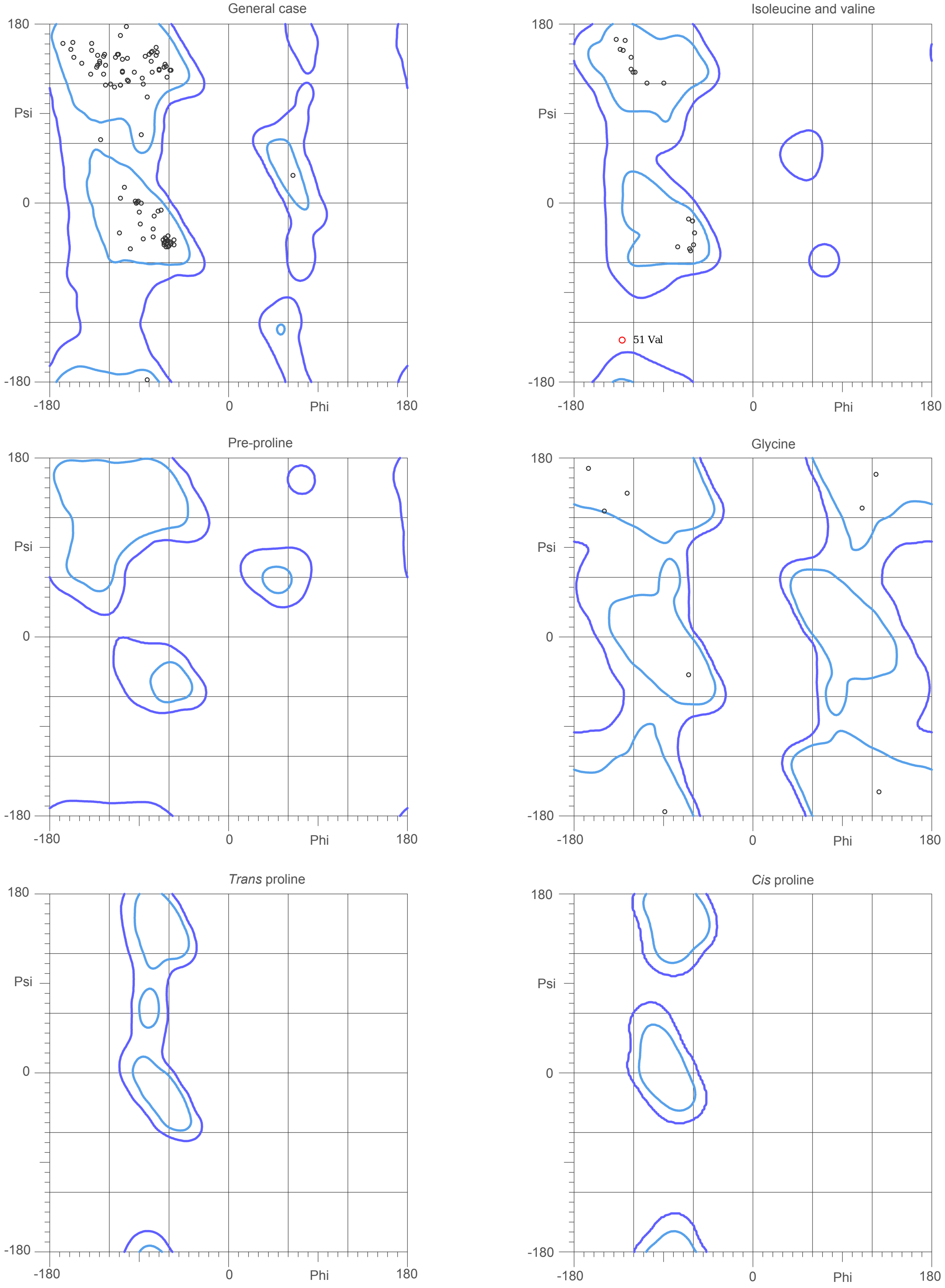

98.3% (117/119) of all residues were in favored (98%) regions.
99.2% (118/119) of all residues were in allowed (>99.8%) regions.

There were 1 outliers (phi, psi):
51 Val (-132.8, -138.8)

MolProbity Ramachandran analysis

fold_2025_11_08_10_19_model_0.pdb, model 1

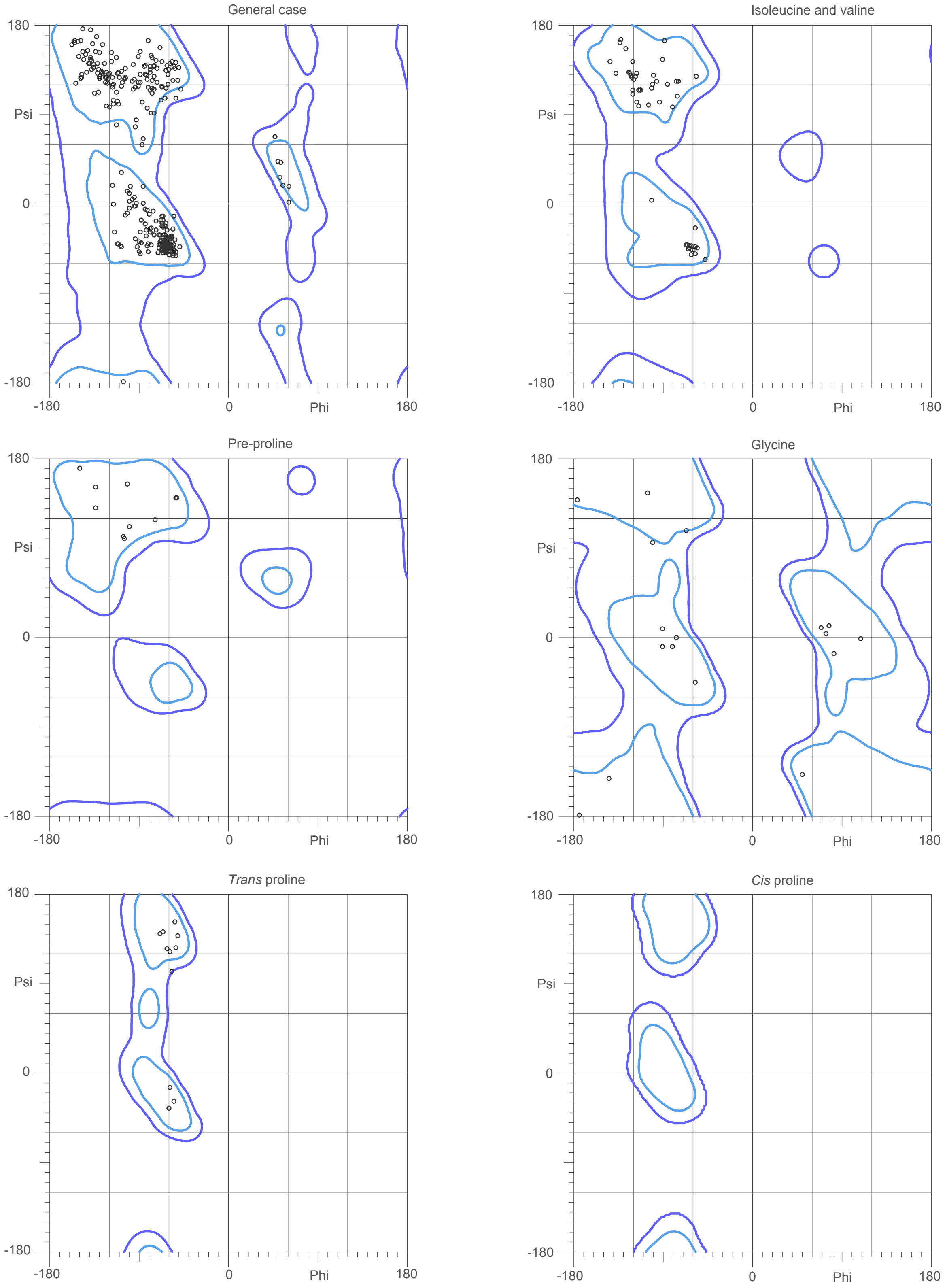

97.4% (380/390) of all residues were in favored (98%) regions.
100.0% (390/390) of all residues were in allowed (>99.8%) regions.

There were no outliers.
